# Colistin in Pig Production: Chemistry, Mechanism of Antibacterial Action, Microbial Resistance Emergence, and One Health Perspectives

**DOI:** 10.3389/fmicb.2016.01789

**Published:** 2016-11-11

**Authors:** Mohamed Rhouma, Francis Beaudry, William Thériault, Ann Letellier

**Affiliations:** ^1^Chaire de Recherche Industrielle du CRSNG en Salubrité des Viandes, Faculté de Médecine Vétérinaire, Université de Montréal, Saint-HyacintheQC, Canada; ^2^Groupe de Recherche et d’Enseignement en Salubrité Alimentaire, Faculté de Médecine Vétérinaire, Université de Montréal, Saint-HyacintheQC, Canada; ^3^Centre de Recherche en Infectiologie Porcine et Avicole, Faculté de Médecine Vétérinaire, Université de Montréal, Saint-HyacintheQC, Canada; ^4^Groupe de Recherche en Pharmacologie Animale du Québec, Faculté de Médecine Vétérinaire, Université de Montréal, Saint-HyacintheQC, Canada

**Keywords:** colistin, pig, resistance, *E. coli*,, mcr-1 gene, humans, One Health

## Abstract

Colistin (Polymyxin E) is one of the few cationic antimicrobial peptides commercialized in both human and veterinary medicine. For several years now, colistin has been considered the last line of defense against infections caused by multidrug-resistant Gram-negative such as *Acinetobacter baumannii, Pseudomonas aeruginosa*, and *Klebsiella pneumoniae*. Colistin has been extensively used orally since the 1960s in food animals and particularly in swine for the control of *Enterobacteriaceae* infections. However, with the recent discovery of plasmid-mediated colistin resistance encoded by the *mcr-1* gene and the higher prevalence of samples harboring this gene in animal isolates compared to other origins, livestock has been singled out as the principal reservoir for colistin resistance amplification and spread. Co-localization of the *mcr-1* gene and Extended-Spectrum-β-Lactamase genes on a unique plasmid has been also identified in many isolates from animal origin. The use of colistin in pigs as a growth promoter and for prophylaxis purposes should be banned, and the implantation of sustainable measures in pig farms for microbial infection prevention should be actively encouraged and financed. The scientific research should be encouraged in swine medicine to generate data helping to reduce the exacerbation of colistin resistance in pigs and in manure. The establishment of guidelines ensuring a judicious therapeutic use of colistin in pigs, in countries where this drug is approved, is of crucial importance. The implementation of a microbiological withdrawal period that could reduce the potential contamination of consumers with colistin resistant bacteria of porcine origin should be encouraged. Moreover, the management of colistin resistance at the human-pig-environment interface requires the urgent use of the One Health approach for effective control and prevention. This approach needs the collaborative effort of multiple disciplines and close cooperation between physicians, veterinarians, and other scientific health and environmental professionals. This review is an update on the chemistry of colistin, its applications and antibacterial mechanism of action, and on *Enterobacteriaceae* resistance to colistin in pigs. We also detail and discuss the One Health approach and propose guidelines for colistin resistance management.

## Introduction

Antibiotics in the polymyxin family include five different chemical compounds (polymyxins A, B, C, D, and E) (Falagas et al., 2005; Gallardo-Godoy et al., 2016), of which polymyxin B and colistin (also called polymyxin E) are the only two polymyxins used clinically (Landman et al., 2008; Cassir et al., 2013). For humans, two forms of colistin are commercially available: colistin methanesulfonate sodium (CMS) for parenteral use and aerosol therapy; and colistin sulfate (CS) for oral and topical use (Li et al., 2006; Brink et al., 2014). Colistin is used in human medicine for the treatment of infections due to multidrug-resistant (MDR) Gram-negative bacteria (GNB) such as *Pseudomonas aeruginosa, Acinetobacter baumannii, Klebsiella pneumoniae*, and carbapenemase*-*producing *Enterobacteriaceae* (Velkov et al., 2009; Azzopardi et al., 2013) and is used as a last-resort treatment option against these infections (Falagas and Rafailidis, 2008; Biswas et al., 2012). Recently, the World Health Organization (WHO) and several government agencies such as Health Canada have reclassified colistin in the category of very high importance in human medicine (WHO, 2011; Government of Canada, 2014). Colistin’s mechanism of action is mainly related to its attachment to the lipopolysaccharides (LPSs) of GNB, leading to membrane-permeability disturbance and cell death (Falagas and Rafailidis, 2008; Biswas et al., 2012).

Colistin sulfate is the only form of colistin approved in pig production in some countries for the control of *Enterobacteriaceae* infections, particularly for those caused by *Escherichia coli* (Guyonnet et al., 2010; Rhouma et al., 2016a). Since its introduction on the market in the 60s, colistin was used in pig production in several countries with different purposes; therapeutically, prophylactically, and even for growth promotion (Katsunuma et al., 2007; Rhouma et al., 2016a). Interestingly, in the late 2000s and after decades of colistin use in swine, several studies began reporting a significant resistance rate of *Enterobacteriaceae* to colistin in pigs (Harada et al., 2005; Enne et al., 2008; Lu et al., 2010; Rhouma et al., 2016a). The most common mechanism of colistin resistance in *E. coli* and *Salmonella* involves a modification of the lipid A portion of LPS through the addition of phosphoethanolamine (PEtN) and/or a 4-amino-4-deoxy-L-arabinose (L-Ara4N), which reduces its binding to colistin and leads to bacterial resistance (Bergen et al., 2012; Olaitan et al., 2014). This chromosomal mechanism of colistin resistance is the result of the activation of the two-component systems (TCSs) PhoP/PhoQ and PmrA/PmrB by specific mutations or environmental stimuli leading to an overexpression of LPS-modifying genes (Olaitan et al., 2014). However, several studies have reported the isolation of colistin resistant *E. coli* strains in the absence of chromosomally encoded mechanisms (Olaitan et al., 2015; Quesada et al., 2015). At the end of 2015, researchers identified a stable plasmid mediated *mcr-1* gene encoded for phosphoethanolamine transferase conferring resistance to colistin in some GNB isolated from food animals, raw meat, and humans in several countries (Liu et al., 2016; Rhouma et al., 2016a). The discovery of this horizontal mechanism of colistin resistance raised alarm bells about the impact of colistin use on colistin resistance spread in animal production, especially in swine. In fact, the link between pigs and humans in terms of colistin resistant *E. coli* strain transfer following direct contact has recently been confirmed (Olaitan et al., 2015). These findings have led to a serious fear about the possible loss of colistin effectiveness in the treatment of MDR-GNB in humans. Hence, it is urgent to establish close cooperation between physicians, veterinarians, and countries to ensure judicious use of colistin in both veterinary and human medicine. The application of the One Health concept could be a solution for the management of colistin resistance in the human-pig-environment interface.

This review is an update on colistin chemistry, its applications and antibacterial mechanism of action, and on *Enterobacteriaceae* resistance in pigs. We also detail and examine the One Health concept to arrive at proposed guidelines for rational use of colistin in swine and humans and to find ways to prevent bacterial resistance spread in the human-pig-environment interface.

Please refer to our recent review for rates of colistin resistance in pigs, the possible link between colistin pharmacokinetic/pharmacodynamic (PK/PD), and colistin use and *Enterobacteriaceae* resistance emergence in swine (Rhouma et al., 2016a).

## Chemical Structure of Colistin and its Antibacterial Mechanism of Action

### Colistin Chemical Structure

**FIGURE 1:**
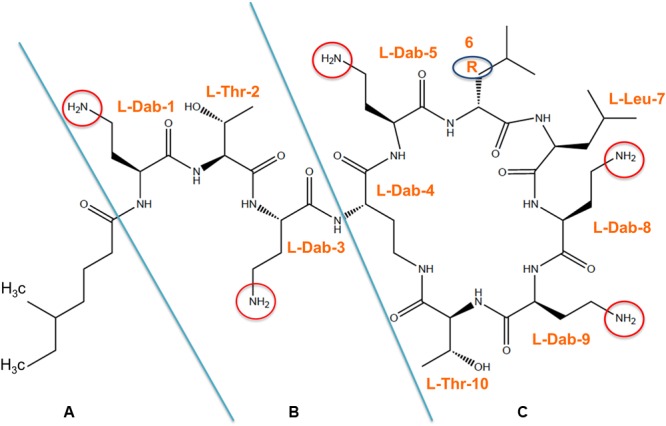
**Chemical structure of colistin is composed of three parts:(A)** hydrophobic acyl tail, **(B)** linear tripeptide segment **(C)** hydrophilic, heptapeptide ring. Arabic numeral indicates the position of amino acids on the structure and the reactive amino groups are encircled. R6: D- phenylalanine in polymyxin B or D- leucine in polymyxin E (colistin).

Colistin (polymyxin E) is a polymyxin antibiotic produced by *Paenibacillus polymyxa* var *colistinus* (Tambadou et al., 2015) consisting of a cyclic heptapeptide ring with three positively charged amine groups, a tail tripeptide moiety with two positively charged amine groups, and a hydrophobic acyl chain tail (**Figure 1**; Li et al., 2006; Bergen et al., 2012; Azzopardi et al., 2013; Dijkmans et al., 2015; Rhouma et al., 2015). Colistin is an amphipathic molecule, with hydrophobicity mainly attributable to the fatty acyl moiety and hydrophilicity due to the five L-diaminobutyric acid (L-Dab) amino groups (Li et al., 2006). The L-Dab molecules are positively charged in positions 1, 3, 5, 8, and 9 (**Figure 1**). These amino groups are responsible for the electrostatically interaction between colistin and the lipid A portion of LPS molecules of GNB and play a central role in the bactericidal activity of colistin (Azzopardi et al., 2013). The polymyxins family includes five chemically distinct compounds (polymyxins A–E) and only colistin (polymyxin E) and polymyxin B have been used in clinical practice (Dijkmans et al., 2015). Polymyxin B and colistin share a similar primary sequence with the only difference being one amino acid in position 6 in which D- phenylalanine in polymyxin B is replaced by D- leucine in colistin (**Figure 1**; Li et al., 2006; Velkov et al., 2009; Biswas et al., 2012; Yoshino et al., 2013; Gallardo-Godoy et al., 2016).

Two different forms of colistin are available commercially: CS, which is administered either orally for bowel decontamination or topically as a powder for skin infections, and CMS, which is commonly administered intravenously and used exclusively in human medicine (Michalopoulos et al., 2011; Bergen et al., 2012). Both can be delivered by inhalation (Li et al., 2006). CS is the only active ingredient of the polymyxin family and is approved in some countries for the control of *Enterobacteriaceae* infections in pigs (Official Journal of the European Union, 2010; Rhouma et al., 2016a; Wan et al., 2016) and is used mostly in monotherapy or sometimes in combination with other substances. Researchers found at least thirty different components in commercially available colistin, 13 of which were separated using the isocratic liquid chromatography (LC) method (Orwa et al., 2000). The two major components of colistin are colistin A (polymyxin E1) and colistin B (polymyxin E2), which differ only in the fatty acid side chain (Orwa et al., 2000). In fact, colistin A and colistin B are acylated by (S)-6-methyloctanoic acid and (S)-6-methylheptanoic acid, respectively (Li et al., 2006). The proportion of these two major components in commercial products differs between the different pharmaceutical preparations of colistin available on the market (Bergen et al., 2012; Brink et al., 2014). This could be due to the fact that colistin is a natural product produced by fermentation, so its composition can vary considerably between manufacturers (Brink et al., 2014). In fact, no pure colistin A and B reference standards are commercially available (Dotsikas et al., 2011) and no certificates of analysis that include chemical characterization are available in veterinary medicine to adequately establish the purity of the marketed CS formulations (Rhouma et al., 2016a). CS is a polypeptide antibiotic with a chemical structure characterized by the presence of multiple peptide bonds documented to predispose CS to chemical and enzymatic degradation (Chihara et al., 1973; Rhouma et al., 2015). In fact, in pig simulated gastric fluid (SGF), CS led to the formation of degradation products that have a significant antimicrobial activity compared to non-degraded CS (Rhouma et al., 2015).

### Colistin Antibacterial Mechanism of Action on *Enterobacteriaceae* in Pigs

Colistin has a narrow antibacterial spectrum with an effect limited to GNB; Gram-positive bacteria do not contain LPS in their cell wall and, as a consequence, are excluded from the spectrum of activity of polymyxins (Dijkmans et al., 2015).

The initial target of colistin is the LPS component of the outer membrane (OM) of GNB. The most documented steps of colistin antibacterial activity are described below (Hancock, 1997; Powers and Hancock, 2003; Velkov et al., 2009; Biswas et al., 2012; Deris et al., 2014b; Martis et al., 2014; Nation et al., 2014; Yu et al., 2015).

(1) Colistin initially binds to LPS and specifically to lipid A, a key component of the LPS, through electrostatic interaction between positively charged Dab residues of colistin and the negatively charged phosphate groups of lipid A. Lipid A plays a crucial role in the control of bacterial permeability (Velkov et al., 2009).
(2) Colistin competitively displaces divalent cations calcium (Ca^2+^) and magnesium (Mg^2+^) that normally stabilize the LPS and as a consequence the three-dimensional structure of the LPS is altered. In fact, colistin has affinities for LPS that are at least three times higher than those for divalent cations (Hancock, 1997).
(3) Colistin causes an expansion of the OM monolayer by the insertion of its hydrophobic terminal fatty acyl chain or the D-Leu^6^ -L-Leu^7^ segment into the OM.
(4) Colistin leads to a permeabilization of the OM by the formation of destabilized areas through which colistin will transit the OM via a self-promoted uptake mechanism (Hancock and Scott, 2000; Straus and Hancock, 2006). This mechanism explains how colistin acts in synergy with conventional antibiotics (Hancock, 1997). In fact, hydrophilic antibiotics such as rifampicin, vancomycin, meropenem, β-lactam, tigecycline, and gentamicin can work synergistically due to this disruption of membrane integrity by colistin (Bolla et al., 2011).
(5) Colistin destroys the physical integrity of the phospholipid bilayer of the inner membrane (IM) through membrane thinning by straddling the interface of hydrophilic head groups and fatty acyl chains (Velkov et al., 2009).
(6) This leads to IM lysis, leakage of intracellular contents and cell death.

Colistin also exerts an anti-endotoxin activity because it binds to the lipid A component of LPS (Falagas et al., 2005; Şentürk, 2005). In this way, colistin prevents endotoxin’s ability to induce shock through the release of cytokines such as tumor necrosis factor-alpha (TNF-α) and IL-8 (Şentürk, 2005; Baeuerlein et al., 2009).

It should be stressed here again that colistin’s antibacterial mechanism of action based on membrane lysis death was the most documented explanation for the effectiveness of this antibiotic in the treatment of GNB infections. However, its ultimate mechanism of action is still unknown (Biswas et al., 2012; Nation et al., 2014). Other mechanisms of polymyxin bactericidal activity have been proposed such as a vesicle–vesicle contact pathway (Cajal et al., 1996; Clausell et al., 2007) and a hydroxyl radical death pathway (Sampson et al., 2012;Yu et al., 2015). The vesicle–vesicle contact antimicrobial mechanism described involves the polymyxin B molecule with a hydrophobic acyl tail that can enter into and cross the OM and induce a lipid exchange between leaflets of the IM and OM; this leads to membrane osmotic instability due to the change in the phospholipid composition, thereby inducing cell lysis (Clausell et al., 2007). However, this mechanism of action has not been studied with colistin. It has been shown that polymyxin B and colistin exert a rapid antimicrobial activity against the sensitive and multidrug-resistant isolates of *A. baumannii* and *E. coli* through hydroxyl radical production by the Fenton reaction (Sampson et al., 2012), leading to the formation of hydroxyl radicals through the reduction of hydrogen peroxide by ferrous iron (Fe^2+^). The production of this reactive oxygen species (ROS) might lead to oxidative damage in the bacterial DNA, proteins, and lipids and cause cell death (Sharma et al., 2016). However, this feature of colistin has not yet been evaluated in clinical practice. Most recently, it was shown that colistin was able to inhibit the vital respiratory enzyme NADH-quinone oxidoreductase in the bacterial IM of GNB (Deris et al., 2014a). This mechanism was regarded as a secondary mode of action of polymyxins.

## Pharmacokinetics and Pharmacodynamics (PK and PD) of Colistin in Pigs

### Clinical PK and PD Studies of Colistin in Pigs

Unlike for human medicine, only a few studies have been conducted in pigs to evaluate the PK of colistin following oral (Guyonnet et al., 2010; Rhouma et al., 2015, 2016b) or intramuscular (IM) administration (Lin et al., 2005; Tang et al., 2009; He et al., 2011; **Table 1**). These studies were performed using CS, since this is the only form of colistin approved in swine medicine, and were conducted in healthy pigs. It is reasonable to think that the PK can be different in sick animals. The oral CS PK data in pigs were obtained using either a high-pressure liquid chromatography (HPLC) assay (Guyonnet et al., 2010) or a LC coupled with the tandem mass spectrometry (HPLC–MS/MS) method (Rhouma et al., 2015, 2016b). CS PK data in pigs after parenteral administration were obtained using mostly microbiological assays (Lin et al., 2005; Tang et al., 2009; **Table 1**); these data should be viewed with caution because of the limited sensitivity of this method and the descriptions of the experiment conditions.

**Table 1:** Colistin sulfate PK data in pigs following its oral or intramuscular administration.

Colistin sulfate route of administration/pigs health status	Dose used (mg/Kg)	Quantification method/LLOQ	Plasma *C*_max_ (ng/mL) Intestine *C*_max_ (mg/Kg)	*T*_max_ (h)	Reference
Oral/clinical healthy	1.2	HPLC	Plasma: NA^∗^	Plasma: NA	Guyonnet et al., 2010
		250 ng/mL	Intestine: 26.97	Intestine: 2	
		0.5 μg/g			
	2.4	HPLC	Plasma: NA^∗^	Plasma: NA	
		250 ng/mL	Intestine: 43.57	Intestine: 1	
		0.5 μg/g			
	4.8	HPLC	Plasma: NA^∗^	Plasma: NA	
		250 ng/mL	Intestine: 91.75	Intestine: 1	
		0.5 μg/g			

Oral/clinical healthy	2.4	LC–MS/MS	Plasma: NA^∗^	Plasma: NA	Rhouma et al., 2015
		20 ng/mL	Intestine: NA	Intestine: NA	

Oral/clinical healthy	2.4	LC–MS/MS	Plasma: 10.3	Plasma: 0.5	Rhouma et al., 2016b
		1 ng/mL	Intestine: NA	Intestine: NA	

Oral/experimental PWD	2.4	LC–MS/MS	Plasma: 122.3	Plasma: 0.5	
		1 ng/mL	Intestine: NA	Intestine: NA	

Oral/clinical healthy	4.8	LC–MS/MS	Plasma: 32.2	Plasma: 0.5	
		1 ng/mL	Intestine: NA	Intestine: NA	

Oral/experimental PWD	4.8	LC–MS/MS	Plasma: 338.3	Plasma: 0.5	
		1 ng/mL	Intestine: NA	Intestine: NA	

IM/clinical healthy	2.4	HPLC	Plasma: 2780	Plasma: 0.5	He et al., 2011
		150 ng/mL	Intestine: NA	Intestine: NA	

IM/clinical healthy	2.5	Microbiological assay	Plasma: NA	Plasma: NA	Tang et al., 2009
			Intestine: NA	Intestine: NA	

IM/clinical healthy	2.5	Microbiological assay	Plasma: 3730	Plasma: 0.5	Lin et al., 2005
			Intestine: NA	Intestine: NA	

IM/clinical healthy	5	Microbiological assay	Plasma: 6400	Plasma: 0.5	Lin et al., 2005
			Intestine: NA	Intestine: NA	

*PWD, post-weaning diarrhea; LLOQ, lower limit of quantitation; C_*max*_, maximum plasma or intestinal colistin concentration; T_*max*_, time at which the C_*max*_ is observed; NA, information not available. ^∗^Concentrations of CS were less than the LLOQ of the method.*

After oral CS administration in pigs and despite the use of a very sensitive analytical method, CS plasma concentrations were very difficult to quantify in healthy pigs (Guyonnet et al., 2010; Rhouma et al., 2015). A concurrent oral challenge of pigs with an ETEC: F4 strain did not increase CS intestinal absorption in a subclinical induction model of post-weaning diarrhea (PWD) (Rhouma et al., 2015). However, in pigs with clinical PWD following an experimental oral challenge with the ETEC: F4 strain, CS plasma concentrations were higher in the challenged groups compared to the unchallenged one (Rhouma et al., 2016b). These studies confirm that colistin is poorly absorbed through pig’s gastrointestinal tract even in infected animals and corroborates the involvement of oral CS administration in exacerbating colistin resistance by exerting selection pressure on pig’s intestinal flora (Rhouma et al., 2016a).

Parenteral CS PK studies in pigs were mainly conducted to study the safety of IM CS administration. The CS intestinal concentrations through the biliary system elimination were not determined following IM administration to assess whether or not colistin exerts a selective pressure on pig’s intestinal microflora after its parenteral administration. There is no available data in the literature concerning the possible renal tubular reabsorption of CS in pigs as previously demonstrated in rats through a carrier-mediated process (Ma et al., 2009); if this is the case, it would justify an extension of the colistin withdrawal period in pigs.

Even though some studies have been able to quantify colistin in the pig’s systemic circulation following its oral administration using very sensitive methods (Rhouma et al., 2016b), these concentrations were very low compared to the Maximum Residue Limits (MRLs) for this molecule in pigs, which supports the short withdrawal period of 1–7 days for oral CS in pigs (Official Journal of the European Union, 2010). In fact, the EMEA Committee for Medicinal Products for Veterinary Use (CVMP) has established the MRLs for colistin in swine: 150, 150, 150, and 200 μg/kg in muscle, liver, fat, and kidney, respectively (Tang et al., 2009). However, no study has been performed in pigs to assess CS degradation product toxicity, and no screening tests are available in the market to detect these products in pig meat (Rhouma et al., 2015). It was shown that *E. coli* experimental infection in pigs increased CS intestinal absorption (Rhouma et al., 2016b), and authors have claimed that this information should be taken into consideration when determining the CS withdrawal period in pigs. Even with intestinal infection, CS systemic concentrations in pigs remain below MRLs, thus adjusting the withdrawal period after *E. coli* infection in pigs should be considered for antibiotics that are characterized by high oral bioavailability.

The potential for the emergence of *E. coli* resistance in pigs during therapy with CS has been shown following its use at the recommended regimen (100,000 IU/kg/day), as demonstrated previously (Rhouma et al., 2016b). In this study, despite a rapid initial reduction in *E. coli* fecal excretion following CS oral treatment, the emergence of CS resistance among commensal *E. coli* was observed starting from the 3rd day of treatment. The selection pressure for CS resistant isolates disappeared after 6 days of CS treatment, and CS resistant *E. coli* strains were isolated 6 days after the last treatment (Rhouma et al., 2016b). This is of significant importance in food safety and public health perspective because this means pigs that are treated with CS and given a 1 day withdrawal period as recommended (Official Journal of the European Union, 2010) are shipped to slaughter with potential colistin resistant *E. coli* in their gut. Therefore, applying a microbiological withdrawal time for CS resistant bacteria in addition to the chemical one is of crucial importance to reduce the risk of passage of these bacteria in pig slaughterhouses to humans through the handling of raw meat or the consumption of undercooked meat.

In order to monitor *E. coli* colistin resistance in pigs subsequent to the therapeutic use of this antibiotic in the treatment of PWD, our team used MacConkey agar medium supplemented with CS at 2 μg/mL, which represents the breakpoint value (Rhouma et al., 2016b). We confirmed that this medium overestimated the number of CS resistant *E. coli* and that the isolation of putative resistant bacteria on this medium requires confirmation by MIC determination using a Mueller–Hinton broth medium. To overcome this problem related to the absence of a selective medium for the screening of colistin resistant bacteria, Nordmann et al. (2016) developed a screening medium that is able to detect intrinsic and acquired polymyxin-resistant bacteria without the need to confirm resistance isolates by MIC determination. The implementation of this medium will facilitate the monitoring of colistin *Enterobacteriaceae* resistance in food-producing animals.

### Perspectives for Colistin (PK/PD) Studies in Pigs

While great advances in colistin research have occurred in the last decade in both human and veterinary medicine (Rhouma et al., 2016a), colistin PK/PD data are very limited in pigs. To successfully combat the development and dissemination of bacterial resistance against this antibiotic in swine, we believe that specific CS clinical PK/PD data are of crucial importance (**Table 2**).

**Table 2:** Topics that should be investigated to ensure judicious use of colistin in pigs.

• Uniform composition and dosing of commercial CS formulations
• Studies to establish specific clinical breakpoints of oral colistin against *Enterobacteriaceae*
• Clinical trials in field conditions to define the optimum dosing strategies, including total daily dose and treatment duration
• Generate more data regarding the PK/PD of colistin in animals with intestinal diseases
• Clinical trials to evaluate the effectiveness of CS treatment at an early stage of disease to reduce colistin quantities used on farms
• Studies to evaluate the effectiveness of CS parenteral formulations and their potential risks on resistance occurrence within intestinal microflora
• Clinical controlled trials to evaluate the potential risks and benefits of combining colistin with other antimicrobial agents
• Studies to elucidate mechanisms of the development of co-resistance to colistin on farms
• Studies to evaluate the efficacy and toxicity of colistin degradation products
• Studies to determine a microbiological withdrawal period for colistin resistant bacteria in addition to the chemical withdrawal period
• Studies to evaluate the expression of *mcr* genes on *Enterobacteriaceae* in pigs

Furthermore, the recent discovery of a plasmid mediated *mcr-1* gene encoding for *Enterobacteriaceae* colistin resistance in farm animals and in humans (Liu et al., 2016) has prompted several regulatory agencies such as the European Medicines Agency (EMA) to re-evaluate colistin in farm animals (European Medicines Agency, 2016a). More data on colistin PK/PD will be essential to ensuring judicious use of colistin in pigs (**Table 2**).

It should be stressed here again that the CS commercially available is obtained by a bacterial fermentation process (Brink et al., 2014; Tambadou et al., 2015). Consequently, its composition may vary between commercially available CMS products (He et al., 2013), although no study in veterinary medicine has verified this variability. In addition, the unit of CS dosing in pig production is not standardized; some practitioners use international units whereas others use milligrams per kg of body weight (Ungemach et al., 2006; Guyonnet et al., 2010; Trauﬄer et al., 2014; Rhouma et al., 2016a). We believe that the standardization of CS composition and dosage in pigs worldwide is critical to ensuring judicious use of this antibiotic, and it would allow comparison between studies in terms of therapeutic efficacy and resistance rate.

Only one study has determined the CS concentrations in clinical healthy intestinal tracts of pigs after a single oral administration of this molecule (Guyonnet et al., 2010). In this study, colistin concentrations were not detectable in fecal samples, from the duodenum to ileum, after 4 h of its oral administration regardless of doses used (25,000, 50,000, or 100,000 IU/kg) (Guyonnet et al., 2010). However, CS is usually administrated in swine medicine to treat sick animals at a dose of 50,000 IU/kg body weight every 12 h for 3–5 days (Official Journal of the European Union, 2010), and the intestinal C_max_ concentrations of colistin were not determined after a repetitive CS oral treatment to justify the efficacy of this therapeutic regimen in the treatment of pig’s diseases associated with *Enterobacteriaceae*. The duration of CS oral treatment in pig farms is far longer than 3–5 days as recommended on product monographs (Chauvin et al., 2002; Van Rennings et al., 2015). Nevertheless, no study in field conditions has evaluated the impact of CS treatment duration on its clinical efficacy in pigs and on bacterial resistance emergence. Our team showed in experimental conditions that 3 days of CS oral treatment of pigs challenged with an ETEC: F4 strain was enough to treat clinical symptoms of PWD in pigs (Rhouma et al., 2016b), and a positive correlation was observed between CS treatment duration and CS selection pressure on commensal *E. coli.*

It has previously been demonstrated that antimicrobial activity is related to inoculum size and stage of infection. Specifically, researchers found that antimicrobial activity may be higher for a lower bacterial inoculum, and treating experimental animals at an early stage of infection reduced both the required dose of antimicrobials and the amplification risk of bacterial resistance in the intestine (Ferran et al., 2011; Vasseur et al., 2014). The impact of the inoculum on the bactericidal activity of colistin has been investigated *in vitro* for some strains of *P. aeruginosa* of human origin (Bulitta et al., 2010). In this study, killing of the susceptible population was 23-fold slower for the 10^9^ CFU and sixfold slower for the 10^8^ CFU than for the 10^6^ CFU. These findings require further investigation in pigs to study the efficiency of an early use of CS in the treatment of infections associated with *Enterobacteriaceae* in swine and to examine the impact of such practice on the resistance amplification risk among pig’s intestinal bacteria and on colistin amounts used at the farm level.

Numerous *in vitro* and *in vivo* studies using colistin and various other antibiotics have provided evidence for increased bacterial killing and decreased emergence of resistance with the use of certain colistin combinations against MDR Gram-negative bacteria (Li et al., 2006; Bergen et al., 2012). Using colistin with other antimicrobial agents (aztreonam, piperacillin, ceftazidime, imipenem, ampicillin-sulbactam, ciprofloxacin, carbapenems, and rifampicin) is the most used combination treatment in human medicine (Li et al., 2006; Martis et al., 2014). Nevertheless, optimal combinations are not defined, and the relative value of a combination may vary between bacterial strains (Clancy et al., 2013).

In swine, and despite the use of some combinations of colistin with other antimicrobial agents (**Table 3**), no study has demonstrated the effectiveness of such association and its role in colistin *Enterobacteriaceae* resistance occurrence.

**Table 3:** Colistin sulfate combination with other antimicrobial agents used in pig production in France (ANSES, 2016).

Combination^∗^	Route of administration	Indications	Withdrawal time (days)
Colistin-Ampicillin	IM	Septicemia, gastrointestinal, respiratory and genitourinary infections	21
Colistin-Amoxicillin	IM	Septicemia, gastrointestinal, respiratory infections	10
Colistin-Erythromycin	Oral	Intestinal infections	21
Colistin-Neomycin	Oral	Intestinal infections	14
Colistin-Oxytetracycline	Oral	Intestinal infections	7
Colistin-Spiramycin	Oral	Intestinal infections	10
Colistin-Trimethoprim	Oral	Intestinal infections	7
Colistin-Ampicillin-Dexamethasone	IM	Septicemia, gastrointestinal, respiratory infections	21

*^∗^Colistin is always used as colistin sulfate. IM, intramuscular.*

Several susceptibility testing methods are used in pigs to determine colistin MIC against bacterial strains of porcine origin (Rhouma et al., 2016a), without specific clinical breakpoints for colistin against *Enterobacteriaceae* after its oral use in swine medicine (Boyen et al., 2010; Richez and Burch, 2016). Such information is of crucial importance for identifying the colistin PD index that is predictive of microbiological efficacy and outcome and to establish the quantitative relationship between PK and PD parameters (Papich, 2014).

Recently, the plasmid-mediated colistin resistance gene *mcr-1* was detected in some Extended-Spectrum-β-Lactamase (ESBL, *bla*_CTX-M_) producing *E. coli* isolates from pigs in Germany and in Vietnam (Falgenhauer et al., 2016; Malhotra-Kumar et al., 2016b). These findings highlight the importance of the active surveillance of colistin resistance in pigs. The suggested strategies to reduce colistin use in pigs should never be associated with an increase in the use of third and fourth generation fluoroquinolones or cephalosporins or the overall use of antimicrobials on farms as claimed in the last report of the EMA (European Medicines Agency, 2016b).

Recommended points of investigation to generate essential PK/PD data for judicious use of colistin in pig production are summarized in **Table 2**.

## Clinical Use and Indications of Colistin in Pig Production

### Indications and Use of Colistin in Pigs

The main indication of colistin in pigs is the treatment of digestive infections caused by *Enterobacteriaceae*, especially for those caused by *E. coli* (Guyonnet et al., 2010). Colistin is widely used for the control of PWD in piglets in Europe (Callens et al., 2012; Kempf et al., 2013). Some epidemiological surveys have been reported that colistin is sometimes used off-label in pig farms to treat infections other than intestinal diseases such as respiratory disease (Chauvin et al., 2002; Catry et al., 2015; Van Rennings et al., 2015). Approximately 99% of colistin use in pig production is carried out orally for mass treatment in intensive husbandry systems (European Medicines Agency, 2016b).

Colistin sulfate is used therapeutically, prophylactically, and even as a growth promoter in swine in some countries (Rhouma et al., 2016a). The CS is not approved in pig production in Canada and in the USA, and this antibiotic is not used as a feed additive for growth promotion in Europe for at least two decades (Kempf et al., 2013). However, CS is used in Canada, in some cases under veterinarian responsibility, as a last resort option for the treatment of PWD in farms with high rates of resistance to aminoglycosides (Rhouma et al., 2016b).

However, the most common use of colistin in pig production worldwide is oral, metaphylactic use (Casal et al., 2007; Trauﬄer et al., 2014). This practice involves treating all animals belonging to the same pen – animals with clinical symptoms as well as clinically healthy ones (Ferran et al., 2011). In its last report, the EMA recommended using colistin only for therapy or metaphylaxis purposes in food-producing animals. All indications for prophylactic use of this molecule should be prohibited and indications of colistin should be restricted only for the treatment of enteric infections caused by susceptible non-invasive *E. coli* (European Medicines Agency, 2016b).

Colistin is used in pigs at the dose of 100,000 IU per kg of body weight for three to five consecutive days and divided into two administrations per day (European Medicines Agency, 2016b). This therapeutic regimen is recommended for colistin veterinary formulations administered in drinking water. However, no recommendation has been made for CS products administered in feed or by an injectable route in pigs. It is important to stress the lack of standardization of therapeutic regimen and its impact on the judicious use of colistin in swine (Catry et al., 2015).

It is difficult to determine the real quantities of colistin used in pig production worldwide because these data vary considerably from one country to another, and sometimes colistin amounts used in pigs in some countries are very high relative to the size of swine herds (European Medicines Agency, 2016b; Mayor, 2016). Even within the same country, quantities of colistin in pigs vary from one survey to another due to the absence of standardized methods for data collection (Casal et al., 2007; Moreno, 2014).

### Combination Therapy

*In vitro* and clinical investigations examining synergism of colistin combined with other antimicrobials in human medicine has been investigated recently and reviewed (Bergen et al., 2015a,b). The ultimate objective of this combination is to overcome the suboptimal exposure and the resistance emergence associated with the use of colistin in monotherapy. Indeed, the combination of colistin with other antibiotics is intended to extend the CS spectrum of activity to cover Gram-positive bacteria and to prevent the emergence of antibiotic resistance (Zhanel et al., 2006). However, a considerable controversy regarding the effectiveness of these combinations to counter the spread of MDR bacteria has been discussed in human medicine (Tamma et al., 2012). Most recently, Lagerbäck et al. (2016) showed that colistin and rifampicin combinations were active *in vitro* against all NDM-1-producing *K. pneumoniae* strains used in their study. However they claimed that such effectiveness should be further explored *in vivo* to be considered for clinical use (Lagerbäck et al., 2016). Parchem et al. (2016) confirmed that colistin combination therapy should be considered in critically ill patients with MDR Gram-negative pneumonia.

In swine, CS is typically used in monotherapy for the oral treatment of infections associated with *Enterobacteriaceae* (Rhouma et al., 2016a). However, there are some commercial formulations where CS is associated with others antimicrobial agents, mostly with β-lactam antibiotics (He et al., 2011) such as ampicillin or amoxicillin (**Table 3**). In fact, it has been shown that the combination of amoxicillin with colistin has a synergy and additive affect *in vitro* against pathogenic *E. coli* of avian origin, without antagonism between the two antibiotics (Hamouda et al., 2011). Colistin combinations were used exclusively for the curative therapy of pig bacterial infections (**Table 3**). Moreover, it has been reported that in the weaning period, colistin was frequently applied in combination therapy with amoxicillin against symptoms of arthritis and/or meningitis and PWD in pigs (Timmerman et al., 2006). Combinations of colistin and amoxicillin plus zinc oxide (ZnO) in the pre-weaning and growing stages in feed were also reported in pigs (Moreno, 2014).

Given the lack of appropriately conducted randomized controlled clinical trials, reliable data on the efficiency of colistin combination use for the treatment of *E. coli* in pigs and its impact on bacterial resistance evolution are very limited or non-existent. In a recent study, Li H. et al. (2016) showed that a combination of CS with bacitracin zinc and chlortetracycline suppressed the increase of *tet* genes in fecal samples of weaned pigs. In this study, the relative fecal abundances of four *tet* genes (*tet*X, *tet*C, *tet*L, and *tet*W) were reduced in pigs treated with a combination of chlortetracycline, bacitracin zinc, and CS compared with the group treated only with chlortetracycline (Li H. et al., 2016). However, in this study no information was reported regarding the evolution of resistance to colistin following the combination use of these antibiotics.

With the lack of solid microbiological evidence on the effectiveness and the impact on bacterial resistance evolution of colistin combination therapy in pigs, the CVMP recommended the withdrawal of marketing authorizations for all veterinary formulations containing colistin in combination with other antimicrobial substances (European Medicines Agency, 2016b).

Heavy metals such as zinc are widely used in pigs, especially for the control of PWD in combination with colistin, and is incorporated into swine feed at levels of 125–3000 mg/kg of feed (Holman and Chénier, 2015). Zinc oxide fed at pharmacological levels reduces diarrhea and mortality and improves growth in pigs (Fairbrother et al., 2005). However, there are two major concerns regarding the use of ZnO in swine. On the one hand, there is environmental pollution because of the high levels of supplementation, and on the other there is co-selection and co-resistance where antibiotic resistance genes (ARGs) are located on the same mobile genetic element as ZnO resistance genes (Holman and Chénier, 2015). To the best of our knowledge, no study has investigated whether or not resistance genes associated with colistin and heavy metals could be carried on the same mobile genetic element. Such information is crucial since ZnO is among the proposed strategies to reduce colistin quantities used for the control of PWD in pig production (European Medicines Agency, 2016b).

In addition to colistin combination therapy used in field conditions (**Table 3**), there are other combinations with this antibiotic that have been used in several scientific studies to evaluate the efficacy of some colistin alternative substances (**Table 4**).

**Table 4:** Colistin combination with other antimicrobial agents in scientific studies conducted in pigs.

Combination	Doses in feed (mg/kg)	Treatment duration (days)	*E. coli* (log10 CFU/g of caecal digesta)	Weight gain (g/d)	Reference
Kitasamycin- Colistin sulfate- Olaquindox	50- 100- 60	14	N/A	307^b^	Li et al., 2008
Kitasamycin- Colistin sulfate- Chlortetracycline	50- 80- 150	35	4.69^a^	505^a^	Li et al., 2012
Kitasamycin - Colistin sulfate	100- 800	19	3.09^a^	367^a^	Wu et al., 2012
Kitasamycin- Colistin sulfate	100- 40	28	N/A	528^a^	Huang et al., 2015
Enramycin- Colistin sulfate- Zinc oxide	200- 200- 2000	28	N/A	787^b^	Kuang et al., 2015

*N/A, not available. a: Statistically significant compared to the control group; b: Not statistically significant compared to the control group.*

These studies (**Table 4**) that evaluated the therapeutic efficacy of colistin combination therapy in pigs were carried out in China and focused primarily on clinical effectiveness not the emergence of antimicrobial resistance. Therefore, no information was available concerning the evolution of colistin bacterial resistance subsequent to the use of these combination therapies in swine, and we do not know whether these combinations are used in practice on pig farms in China.

## Mechanisms of *Enterobacteriaceae* Resistance to Colistin

Owing to an excessive use of colistin in pig production for many decades, several studies conducted with swine reported the isolation of *E. coli* and *Salmonella* strains with high percentages of resistance to colistin (Rhouma et al., 2016b). In the present review we will detail the mechanisms of resistance to colistin for *Salmonella* and *E. coli*, due to the importance of these two bacteria in both swine and human health.

### Chromosomal Resistance

An initial and essential step in colistin action on GNB is the electrostatic interaction between the positively charged peptide of this antibiotic and the negatively charged lipid A of LPS (Deris et al., 2014b). Chromosomal resistance to colistin in *Salmonella* and *E. coli* is most often mediated by modifications of LPS, which result in alterations in the target and reduced binding of the antimicrobial (Biswas et al., 2012). Changes in LPS consist in a modification of lipid A with the addition of a 4-amino-4-deoxy-L-arabinose (L-Ara4N) and/or phosphoethanolamine (PEtn). These molecules reduce the net negative charge of LPS and as a consequence increase the resistance to colistin (Needham and Trent, 2013). In *Salmonella* and *E. coli*, the biosynthesis of l-Ara4N and/or PEtn is mediated by PmrA/PmrB and PhoP/PhoQ two-component response regulators and sensor kinase systems (Falagas et al., 2010). In fact, the PhoPQ and PmrAB TCSs in *Salmonella* and *E. coli* have been reviewed extensively elsewhere (Needham and Trent, 2013; Olaitan et al., 2014). A brief overview is provided here with a focus on the more recent discoveries.

PmrB and PhoQ are sensor cytoplasmic membranes activated respectively by high concentrations of Fe^3+^ and low pH and by low concentrations of Mg^2+^ and Ca^2+^ or certain antimicrobial peptides (McPhee et al., 2003; Rubin et al., 2015). In colistin resistant *Salmonella*, apart from an environmental stimuli such as low Mg^2+^ concentration, a mutation in the PmrA/PmrB and/or PhoP/PhoQ TCS is the major mechanism involved in LPS modification (Olaitan et al., 2014). In *Salmonella*, PhoPQ further influences lipid A modification by activating the PmrAB system through the activation of PmrD (Kato et al., 2012). However, it was proposed in *E. coli* that the two systems are not coupled because PmrD does not activate the PmrA/PmrB system (Winfield and Groisman, 2004). This hypothesis was initially justified by a high divergence between the *Salmonella* and *E. coli* PmrD proteins (Winfield and Groisman, 2004). However, it was later found that *E. coli* PmrB possesses higher phosphatase activity that exceeds the same activity of the *Salmonella* homolog, and the replacement of the *E. coli pmrB* gene with the *Salmonella* homolog was able to render *E. coli* resistant to polymyxin under PmrD-inducing conditions with low concentrations of Mg^2+^ (Chen et al., 2011). Moreover, it has been demonstrated that the sRNA MgrR of *E. coli* was also involved in the regulation of lipid A modification (Moon and Gottesman, 2009). Most recently, Rubin et al. (2015) have shown that in *E. coli*, another unknown bacterial system activates PmrD under low Mg^2+^ conditions to promote lipid A modification, even in the absence of PhoPQ.

Mutations in TCS corresponding to *E. coli* and *Salmonella* can cause their constitutive over expression, leading to permanent modification of lipid A by L-Ara4N and PEtN (Olaitan et al., 2014). Recently, various mutations have been identified in both *pmrA* and *pmrB* genes of colistin-resistant *E. coli* isolated from healthy pigs and pigs with intestinal disease (**Table 5**). Mutations in the PmrAB TCS are mostly involved in the development of resistance to colistin in *E. coli* (Quesada et al., 2015).

**Table 5:** Mutations in two-component systems conferring resistance to colistin in *E. coli* of pig origin.

Bacteria	Health status/samples	Gene	Mutation in aa	MIC (mg/L)	Reference
*E. coli*	Clinical healthy/feces	*pmrA*	S39I	4	Quesada et al., 2015
			R81S		
*E. coli*	Clinical healthy/feces	*pmrB*	V161G	4	Quesada et al., 2015
*E. coli*	Experimental PWD/feces	*pmrA*	G53R	8	Thériault, 2015
*E. coli*	Experimental PWD/feces	*pmrB*	T156M	8	Thériault, 2015

*aa, amino acid; PWD, post-weaning diarrhea; MIC, minimum inhibitory concentration.*

For PmrA, mutations mostly occurred in the phosphate acceptor domain, while for PmrB, mutations most commonly occurred in the kinase domain (Quesada et al., 2015).

Of note, regardless of the mutation location in PmrA or PmrB genes, there was no association with a difference in MIC of these colistin resistant *E. coli* strains (**Table 5**).

Despite the fact that polymorphism in the PmrAB system has been reported *in vitro* in *Salmonella* (Sun et al., 2009), Quesada et al. (2015) did not detect any of the protein polymorphisms of PmrA and PmrB sequences in colistin resistant *Salmonella* isolates from swine lymph nodes. However, the polymorphism of genes encoding the PhoPQ system in colistin-resistant *Salmonella* has not been investigated in this study. Recently, an in-depth investigation of these *Salmonella* isolates showed that 100% of them harbored the plasmid carrying the *mcr-1* gene (Quesada et al., 2016).

Furthermore, the effects of colistin resistance on virulence and on *in vitro* and *in vivo* fitness costs have been extensively studied in other GNB such as *A*. *baumannii* and *K*. *pneumoniae* (Beceiro et al., 2014; Choi and Ko, 2015). A study of the fitness costs of colistin resistant *Salmonella* pmrAB mutants *in vitro* and in a mouse model showed low fitness costs for these strains (Sun et al., 2009). Nevertheless, to the best of our knowledge no study has followed the fitness costs of colistin resistant *E. coli* mutants. Many studies have discussed the factors affecting the fitness cost of colistin resistance, including growth retardation, impaired virulence, increased susceptibility to other antibiotics, and substantially reduced clinical invasiveness (López-Rojas et al., 2011; Pournaras et al., 2014). In swine, it has been reported that oral colistin treatment is accompanied by a selection pressure on the colistin resistant *E. coli* commensal population (Rhouma et al., 2016b). Further investigations are required to study the fitness costs of colistin resistant *E. coli* and *Salmonella* of porcine origin.

### Plasmid-Encoded Colistin Resistance

Before November 2015, several studies in human and in swine medicine confirmed the isolation of *E. coli* isolates confirmed resistant to colistin without having a mutation in *pmrA* and/or *pmrB* genes (Olaitan et al., 2015; Quesada et al., 2015). The discovery for the first time in early November 2015 in China of a plasmid mediated colistin resistance-1 (MCR-1) protein in *Enterobacteriaceae* (Liu et al., 2016) has provided explanation for the other potential colistin resistance mechanisms in GNB. Initially, this plasmid was considered to be a phenomenon relegated to China (Paterson and Harris, 2016), however the *mcr-1* gene was soon after isolated in several countries on 4 continents: Asia, Africa, Europe, and the Americas (Rhouma et al., 2016a; Schwarz and Johnson, 2016; Skov and Monnet, 2016).

Very recently, in June 2016, a novel plasmid-mediated colistin resistance gene, *mcr-2*, was identified in colistin resistance *E. coli* isolates from porcine and bovine origin in Belgium (Xavier et al., 2016b). The *mcr-2* gene was detected with higher prevalence than of *mcr-1* gene among colistin-resistant *E. coli* of porcine origin.

MCR-1 and MCR-2 proteins showed 80.65% of identity and are members of the phosphoethanolamine transferase enzyme family that promotes the addition of a phosphoethanolamine group to lipid A, leading to a decreased affinity of colistin for the LPS (Liu et al., 2016; Xavier et al., 2016b). In Liu et al. (2016) study, the *mcr-1* associated plasmid, designated pHNSHP45, is approximately 64 Kb in size and is an IncI2-like plasmid that harbors a predicted 83 open reading frames (ORFs) with a G+C content of 42.7%. The plasmid pHNSHP45 carrying *mcr-1* gene was initially isolated in July 2013 from an *E. coli* strain recovered from a pig farm (Shanghai, China) and showed resistance to most antibiotic families except the carbapenems (Liu et al., 2016). Subsequently, *mcr-1* has been reported in different plasmid incompatibility groups from different animal species, including IncHI2 (200–290 Kb), pVT553 (62 Kb), IncX4 (30 Kb), and IncP (79 Kb) plasmids in *E. coli* from broilers poultry, bovine, and swine origin (Anjum et al., 2016; Falgenhauer et al., 2016; Malhotra-Kumar et al., 2016a; Perreten et al., 2016; Veldman et al., 2016) and IncX4 (30 Kb) plasmids in *Salmonella* from chicken and turkey meat (Veldman et al., 2016; Webb et al., 2016). Xavier et al. (2016a) isolated the *mcr-1* gene in pKP81-BE plasmid (91 Kb) from colistin resistant *E. coli* of porcine origin. The pKP81-BE plasmid showed a G+C content of 44.9% and belonged to IncFII incompatibility type with 4% similarity compared to pHNSHP45. These findings showed that *mcr-1* has horizontally transferred to other plasmid types, leading to an increase in its target bacterial range (Li A. et al., 2016; Tse and Yuen, 2016).

The *mcr-2* associated plasmid, designated pKP37-BE, is approximately 35 Kb in size and is an IncX4 incompatibility type, with a G+C content of 41.3%, and did not carry any other resistance genes (Xavier et al., 2016b).

The *mcr-1* gene has been identified in *Enterobacteriaceae* derived from humans, food, farm animals (Liu et al., 2016), vegetables (Zurfuh et al., 2016), the environment including water (Petrillo et al., 2016), and even wild migratory bird (Ruzauskas and Vaskeviciute, 2016). The *mcr-1* gene has also been identified in several multidrug resistant bacteria such as ESBL producing and carbapenemase-producing *E. coli* of chicken and swine origin (Falgenhauer et al., 2016; Yao et al., 2016). In colistin resistant *E. coli*, a co-localization of *mcr-1* and *bla*_CTX-M_ genes on a unique IncHI2-type plasmid was also reported in chickens (Grami et al., 2016; Sun et al., 2016) and in calves (Haenni et al., 2016). The co-localization of *mcr-1* with an ESBL gene on a conjugative plasmid increases the possibility of bacterial resistance to colistin and of broad-spectrum cephalosporins being maintained, even without the use of theses antibiotics in food animals. This finding poses significant challenges for successful clinical treatment of GNB and for resistance control strategies in both veterinary and human medicine. Veldman et al. (2016) reported for the first time a chromosomally located *mcr-1* gene in two colistin resistant *E. coli* isolated from veal calves. In this study, the *mcr-1* gene was associated with the insertion sequence (IS) IS*Apl1*-*mcr-1* (or an *mcr-1*-containing mobile element) located immediately upstream of *mcr-1*, as also reported in plasmid pHNSHP45 (Liu et al., 2016). IS*Apl1* is a member of the IS*30* family, which was initially identified in *Actinobacillus pleuropneumoniae* (Tegetmeyer et al., 2008). The presence of this IS in association with the *mcr-1* gene strongly suggests that this gene is able to translocate to the chromosome and to different plasmid backbones – as well as between bacterial strains. Furthermore, the *mcr-2* gene was associated with an IS of the IS_1595_ superfamily (Xavier et al., 2016b).

In swine, to the best of our knowledge, the plasmid-borne *mcr-1* gene has been observed in at least 2 enterobacterial species, *E. coli* and *Salmonella*, in ∼12 countries on four different continents (Rhouma et al., 2016a; Schwarz and Johnson, 2016). Pig-to-human transmission of MCR-1 colistin resistance has already been reported (Olaitan et al., 2015, 2016a), raising serious concerns about the consequences of the use of this antibiotic in pig productions on human healthcare.

In pigs, the *mcr-1* gene was isolated mainly from colistin resistant *E. coli* strains with variable prevalence between countries; China (20.6%), Vietnam (22%), Belgium (13.2%), Brazil (2%), Spain (0.68%), Germany (0.51%), and France (0.50%) (Falgenhauer et al., 2016; Fernandes et al., 2016; Liu et al., 2016; Malhotra-Kumar et al., 2016a; Nguyen et al., 2016; Perrin-Guyomard et al., 2016; Quesada et al., 2016). Most recently, in the USA pig production, the *mcr-1* gene was identified for the first time in a colistin resistant *E. coli* strain isolated from a pig from South Carolina (Meinersmann et al., 2016). In these studies, despite using the same technique (PCR) for *mcr-1* gene screening, it is difficult to compare these results between countries because of the lack of data on previous antibiotic treatments in sampled pigs, on the quantities of colistin used at the farm level, on the potential combination of antibiotics with colistin, and on the health status of the pigs. Moreover, there are no published longitudinal studies on pigs that quantify the link between colistin quantities used on farms and the evolution of bacterial resistance against this antibiotic.

Almost all studies conducted on pigs worldwide to screen *mcr-1* gene presence in enterobacterial species reported that colistin resistant strains harboring this gene also showed resistance to one or several classes of antibiotics conventionally used in swine such as: Aminoglycoside, Sulfonamide, Trimethoprim, Tetracycline, Quinolone, Lincosamide, β-lactam, and third generation cephalosporin (Anjum et al., 2016; Falgenhauer et al., 2016; Malhotra-Kumar et al., 2016b; Nguyen et al., 2016). This multi-resistance of *mcr-1* positive *E. coli* strains in pigs was associated with the presence of a *sul3*-containing class 1 integron, In*640*, in the plasmid’s mediated *mcr-1* gene. This integron showed the presence of genes encoding resistance to trimethoprim (*dfrA12*), aminoglycosides (*aadA1a* and *aadA2*), sulfonamides (*sul3*), and phenicols (*cmlA1*) (Xavier et al., 2016a). Furthermore, IncX4 plasmids have been shown to harbor *mcr-1 and mcr-2* genes a swell as ESBL genes (Xavier et al., 2016b).

In the study of Quesada et al. (2016), the *mcr-1* gene was screened and detected in three colistin resistant *Salmonella* strains isolated from 122 lymph nodes and in two colistin resistant *E. coli strains* isolated from 439 swine fecal samples. This study was the first in swine to demonstrate the existence of a plasmid carrying *mcr-1* gene, in addition to a mutation in PmrAB TCS, in two colistin resistant *E. coli* strains. The coexistence of these two colistin resistance mechanisms in *E. coli* was not associated with a difference in the MIC of these strains compared to resistant *Salmonella* strains that expressed only the plasmid carrying *mcr-1* gene (Quesada et al., 2016). It should be stressed here that the *mcr-1* gene found in colistin resistant enterobacterial strains of porcine origin was often associated with low levels of resistance; the MICs of 4 or 8 mg/L observed for most isolates are only 2–4 times higher than the EUCAST clinical breakpoint (2 mg/L) (Anjum et al., 2016; Liu et al., 2016; Quesada et al., 2016). Fernandes et al. (2016) reported the isolation of a colistin-susceptible *E. coli* strain carrying the *mcr-1* gene from the fecal sample of a healthy pig. This finding, suggests that *mcr-1*-positive isolates may be difficult to detect if only the *mcr-1* gene is screened in colistin resistant isolates. Further studies are needed to examine the expression of *mcr-1* gene in *E. coli* and to determinate the promoter and the operon responsible for this expression.

## One Health Perspectives

### Importance of the One Health Concept in Colistin Resistance Management

Currently, colistin is an antibiotic widely used in veterinary medicine, particularly in pigs, for the oral treatment of intestinal infections caused by *Enterobacteriaceae* (Rhouma et al., 2016a). In humans, colistin is used for the treatment of infections caused by MDR-GNB and is considered to be a last-resort antibiotic treatment option for carbapenemase-producing *Enterobacteriaceae* infections (Gurjar, 2015). During the last decade, research on colistin experienced a significant increase, especially regarding the mechanism of resistance of colistin and the optimization of its therapeutic regimen using the PK/PD relationship (Michalopoulos and Falagas, 2011; Olaitan et al., 2014).

**FIGURE 2:**
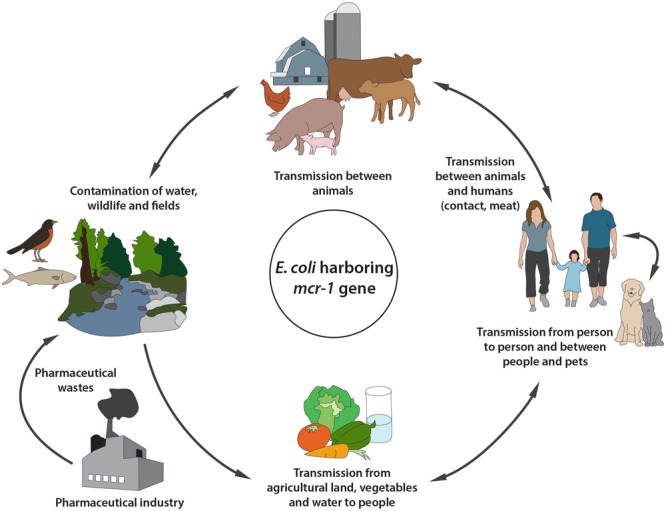
**Circulation of colistin resistant *Escherichia coli* harboring *mcr-1* gene between animals-environment-food and humans**.

Recently, the *mcr-1* gene was isolated from colistin resistant *E. coli* strains from several farm animals: pigs (Rhouma et al., 2016a), piglets (Malhotra-Kumar et al., 2016a), chickens (Shen et al., 2016), cattle (Suzuki et al., 2016), and veal calves (Haenni et al., 2016). A strong similarity was found between the different classes of plasmid carried *mcr-1* genes in these animal productions, and the successful gene-plasmid combination was mainly attributed to the presence of IS*Apl1* upstream in the *mcr-1* gene (Falgenhauer et al., 2016). These findings are in favor of a possible movement of this mobile genetic element between the various animal productions (Falgenhauer et al., 2016; **Figure 2**). In addition to their use in pigs, polymyxins and especially polymyxin B are used in some countries for the treatment of coliform and *Pseudomonas* mastitis in cows (Du Preez, 2000), and this antibiotic is sometime used for this purpose as an extra-label drugs in cattle such as in Canada and in the United States (Smith et al., 2005). Intramammary infusions of 1–2 million units of polymyxin B/quarter gave an efficiency for the treatment of cows with severe cases of coliform mastitis (Smith et al., 2005). Although, some studies have reported the isolation of colistin resistant *E. coli* strains harboring the *mcr-1* gene from cow with mastitis (Suzuki et al., 2016), the role of polymyxin B, used for the treatment of mastitis, in colistin resistance still unknown. Furthermore, colistin is used outside of North America orally in calves and lambs at a dose of 100.000 IU/kg b.w day divided in two identical doses for three consecutive days for the treatment of gastrointestinal diseases caused by GNB (Official Journal of the European Union, 2010). This use could explain the isolation of bacteria resistant to colistin in calves, even despite the lack of data on an preliminary treatment of these animals with colistin (Haenni et al., 2016).

On the other hand, colistin is used in some countries such as China for the control of intestinal infection caused by GNB in chicken, turkeys, rabbits and ducks (Dowling, 2013). Colistin was incorporated into the feed of these animals at the dose of 3.33 mg/kg b.w for turkeys, 3.8 mg/kg b.w for rabbits and chickens, and 20 mg/kg for ducks (Zeng et al., 2010). Colistin was also used in the drinking water in laying hens at the dose of 3.8 mg/ kg b.w (Goetting et al., 2011). Furthermore, colistin is widely used in Europe for the oral treatment of *E. coli* infections in chicken and laying hens at the dose of 75.000 IU/kg b.w day for 3–5 consecutive days in the drinking water (Official Journal of the European Union, 2010; Le Devendec et al., 2015). Although, several studies have confirmed the isolation of bacteria resistant to colistin harboring *mcr-1* gene from avian origin, however, to the best of our knowledge no scientific study has investigated the resistance of GNB to colistin in turkeys, rabbits, and ducks.

In addition, the *mcr-1* gene was also isolated from wild migratory birds such as the European herring gull (*Larus argentatus*) in Lithuania (Ruzauskas and Vaskeviciute, 2016) and the kelp gulls (*Larus dominicanus*) in Argentina (Liakopoulos et al., 2016). The role of these migratory birds in the spread of the *mcr-1* gene between continents should not be underestimated.

The *mcr-1 gene* was identified in resistant *E. coli* strains isolated from environmental samples such as river water (Zurfuh et al., 2016), chicken feed in trough (Yu et al., 2016), and ready-to-eat vegetables (Zurfuh et al., 2016). Therefore, the role of animal manure used in the fertilization of agricultural lands in the environmental dissemination of the *mcr-1* gene needs to be verified. Several studies have reported the isolation of colistin resistant bacteria from pig manure (Hölzel et al., 2010).

In addition, the *mcr-1* gene was identified in resistant *E. coli* strains isolated from food samples such as chicken and pork meat (Liu et al., 2016), ground beef (Mulvey et al., 2016), and retail meats (chicken, pork, and beef) (Kuo et al., 2016). These foods of animal origin represent a major route of contamination with the *mcr-1* gene for slaughterhouse workers and consumers (**Figure 2**).

The gene encoding plasmid-mediated colistin resistance, was also identified in resistant *E. coli* strains isolated from humans with gastroenteritis or wound infections (Doumith et al., 2016; Falgenhauer et al., 2016) and from asymptomatic people (Olaitan et al., 2016a). The *mcr-1* gene was isolated from humans from four continents, showing that plasmid-mediated colistin resistance has already spread worldwide.

It was reported that food animals are the main source of human contamination by the MCR-1 and MCR-2 (Nordmann and Poirel, 2016; Rhouma et al., 2016a; Xavier et al., 2016b). However Ruppé et al. (2016) isolated the *mcr-1* gene in colistin resistant *E. coli* from five children with ages ranging between 2 and 27 months who did not have pets or a history of animal contact. Moreover, despite the fact that colistin is not approved in animal production in the USA, McGann et al. (2016) reported for the first time in the USA, the identification of *mcr-1* gene in a colistin resistant *E. coli* strain cultured from a woman with a urinary tract infection (UTI). However this strain remained susceptible to several other antimicrobial agents (McGann et al., 2016). These findings suggest that *mcr-1* is already widespread in the environment and transmissible via various routes to humans. Thereby, there is also a potential risk of the transfer of *mcr-1* gene from human to animal. However such transfer should be investigated in future studies.

Most recently, *mcr-1-*harboring *E. coli* was isolated from healthy dogs and cats in a pet shop in Guangzhou, China (Zhang, 2016). An interesting finding in this study was that the *mcr-1* gene in colistin resistant *E. coli* was isolated from a worker at this pet shop – and it was the same *E. coli* strain clonally related to those originating from dogs. This finding is in favor of a possible transmission of *mcr-1*-harboring *E. coli* between dogs and humans.

Polymyxins are used in dogs and cats mostly for topical indications (Mateus et al., 2011; De Briyne et al., 2014). In fact, polymyxin B is used in the treatment of canine otitis externa, and it showed synergy with miconazole against *E. coli* and *P. aeruginosa* (Pietschmann et al., 2013). Polymyxin B used also in ophthalmic suspension for the treatment of keratitis in dogs (Beckwith-Cohen et al., 2015). For the treatment of this ophthalmic disease, polymyxin B is commonly associated with other drugs such as neomycin, and dexamethasone (Beckwith-Cohen et al., 2015), or chloramphenicol (Hindley et al., 2016). Furthermore, it was shown that colistin used at the dose of 12. 500 IU/kg IM for 5 days in combination with ampicillin had demonstrated an anti-endotoxic effects in dogs with naturally occurring endotoxic shock (Şentürk, 2005). Despite the isolation of *E. coli* resistant to polymyxins harboring the *mcr-1* gene from dogs and cats, it is difficult to determine the role of polymyxin B administered topically in the exacerbation of colistin resistance in dog’s or cat’s intestine.

Neither the role of waste and contaminants from the pharmaceutical industry nor the role of fish farms has been documented as a source of colistin resistance amplification in the environment. In fact, it has been reported that administration of colistin sulfate with other antibiotics in the diets of fish significantly improved feed conversion and promoted their growth rate (Hao et al., 2014). To the best of our knowledge, no study has documented the isolation of colistin resistant *E. coli* strains or *mcr-1* gene from fish.

Transmission of *mcr-1* gene resistance from animals to humans can take place through a variety of routes (**Figure 2**). Therefore, the management of colistin resistance requires global and coordinated action between the different actors in order to intercept this resistance spread and preserve the efficacy of colistin for the treatment of MDR-GNB in human medicine.

**FIGURE 3:**
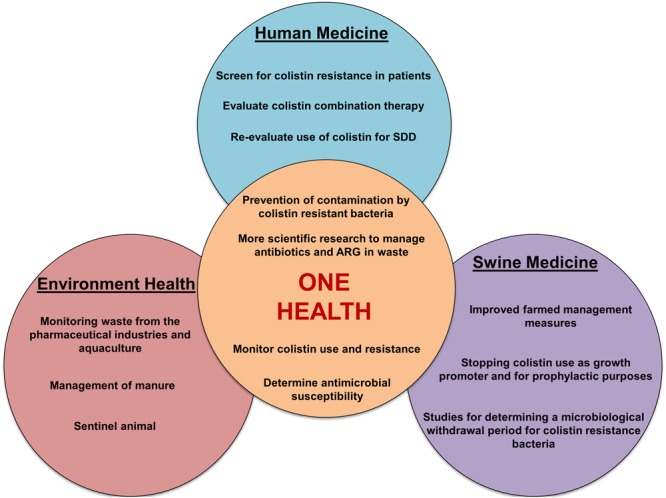
**Schematic representation of various actions to be undertaken to ensure reliable management of colistin resistance in a One Health perspective**. SDD, selective decontamination of the digestive tract; ARG, antibiotic resistance genes.

We believe that the One Health concept is more important than ever to better manage the impact of colistin resistance in human and veterinary medicine. Such a concept needs a global strategy to develop collaborations and interdisciplinary communication between concerned specialists (**Figure 3**).

### Action in Swine Medicine

The use of colistin in swine has contributed to the intensification of modern pig productions by assuring successful weaning, higher animal densities, and most likely helped to reduce economic losses caused by *E. coli* infections such as PWD and edema disease (Rhouma et al., 2016a). Economic gains have come at a considerable cost, which is being borne, in particular, by public health and other stakeholders such as the environment and the animals themselves. In fact, the recent discovery of a plasmid-mediated *mcr-1* gene encoding for colistin resistance in *Enterobacteriaceae* has aroused great concern about the possible loss of colistin effectiveness for the treatment of MDR- GNB in humans. Because of the high rate of isolates carrying the *mcr-1* gene isolated from animals compared to humans, livestock production has been pinpointed as a reservoir of the *mcr-1* determinant (Nordmann and Poirel, 2016), hence the need for rapid action in food animals to prevent the spread of colistin resistance (**Figure 3**). This section will focus on interventions in swine medicine but is applicable to all animal productions where colistin is used.

#### The Use of Colistin as a Growth Promoter

This practice should be banned internationally. In addition to the fact that antimicrobials for growth promotion can generally be purchased without veterinary involvement, low subinhibitory concentrations of antibiotics used to improve animal growth has been shown to promote antibiotic resistance emergence (Aminov and Mackie, 2007; Andersson and Hughes, 2010; Nosanchuk et al., 2014). No recent studies have been able to clearly establish a link between the use of antibiotics as growth promoters and the improvement of animal performance in modern farming conditions with a high level of sanitation (Diarra and Malouin, 2014).

#### The Use of Colistin for Prophylaxis and Metaphylactic Purposes

This usage is involved in the increase of colistin quantities used in pigs and increases its presence as waste in the environment (Rhouma et al., 2016a). Such usage of an antibiotic of very high importance in human medicine should be strictly avoided in swine. Intestinal disease prevention in pigs should be based mainly on livestock preventive management measures (optimal temperature, vaccination, sanitation, housing conditions, applying biosecurity rules, etc.) (Fairbrother et al., 2005; Aarestrup et al., 2008a).

#### The Use of Colistin for Therapeutic Purposes

Nevertheless that colistin is a cheap therapeutic strategy with certain efficacy against Enterobacteria associated disease in swine, it has been shown that the oral use of colistin for the treatment of pigs in an experimental PWD model was associated with a pressure selection on *E. coli* populations (Rhouma et al., 2016b). Therefore, the use of colistin as the first therapeutic choice to treat intestinal infections in pigs should be avoided. The therapeutic alternative to colistin should not be an antibiotic belonging to β-lactam family because of the co-localization of *mcr-1* and ESBL genes in the same mobile genetic element. In addition, in its very recent advice, the EMA required that the reduction of colistin use in farm animals should not be associated with an increase in the consumption of fluoroquinolones, third- and fourth-generation cephalosporins, or the overall use of antimicrobials (European Medicines Agency, 2016b).

#### Requirements for Colistin Therapeutic Use

Clinical diagnoses of the disease by veterinarians and the isolation of pathogen agent linked with the antibiogram tests to determinate bacterial susceptibility to colistin are essential to justifying its therapeutic use. Isolation from the animal husbandry of bacteria harboring the *mcr-1* gene should be considered a strong reason not to use colistin on that farm.

Moreover, the veterinarian should ensure that colistin prescribed is used in farms only for the treatment of sick pigs as recommended; compliance with label instructions (no underdosing or prolongation of dosing interval, withdrawal period) is of paramount importance. Any deviations from the guideline recommendations must be justified and recorded. In this context, extra-label use of colistin in some countries where this antibiotic is not approved in swine such as in Canada, must take place within a valid veterinarian-client-patient relationship. An analysis of the specific situation at farms and a determination that there are no alternatives to this antibiotic for the treatment of this case is required. Research is very important in order to establish a microbiological withdrawal period that could reduce the risk that pigs sent to slaughter contain colistin resistant bacteria or *mcr* genes in their gut.

#### Surveillance and Monitoring of Colistin Use on Farms

Veterinarians should ensure that colistin use targets clinical disease, should consider reduction of its use whenever practical, and should direct management and husbandry issues at the same time. Veterinarians should also consider laboratory examination as a routine practice to evaluate the effectiveness of colistin treatment and to monitor the sensitivity of infectious strains on the farm. Educational and awareness campaigns for employers and pig farmers are essential to generate an understanding that can support the veterinarian to withhold colistin. The professional organization of each country should develop clinical-practice guidelines on the judicious use of colistin. Data on colistin usage in food animals are critically important because they provide a basis for the development of national policies and they guide the risk of colistin resistance management and assess the effect of possible interventions (Aarestrup et al., 2008b). At a minimum, these data should include national use of colistin in kilograms of active ingredient on an annual basis and data should be stratified by animal species (Merle et al., 2012). The OIE and WHO recommend collecting the amount of antibiotics in food animals (WHO, 2004). Finally, the standardization of a data collection method regarding the use of colistin in farms between countries is very important to evaluate the effectiveness of such interventions to manage colistin resistance spread.

#### Monitoring of Colistin Resistance

There are many national antimicrobial resistance monitoring and surveillance programs that already exist and are well-established in many countries (Gelbrand et al., 2015; **Table 6**). Among the principles of the One Health approach is the improved use of existing natural resources and implementation, which includes the monitoring of colistin resistance spread in both human and veterinary medicine. However, regulations and practices vary widely between these surveillance programs and are influenced by the economic and social context of each country (Laxminarayan et al., 2013).

**Table 6:** Examples of antimicrobial resistance monitoring and surveillance programs in some countries.

Countries	Name of surveillance program	Directed by	Target
European Union	The European Antimicrobial Resistance Surveillance System (EARSS)	European Centre for Disease Prevention and Control (ECDC)	Humans
Denmark	The Danish Antimicrobial Resistance Monitoring and Research Program (DANMAP)	Danish Ministry of Food, Agriculture and Fisheries and the Danish Ministry of Health	Humans, animals, and food
Canada	The Canadian Integrated Program for Antimicrobial Resistance Surveillance (CIPARS)	Health Canada	Humans, animals, and meat
United States	National Antimicrobial Resistance Monitoring System (NARMS)^†^	Food and Drug Administration Center for Veterinary Medicine (FDACVM)	Humans, animals, and meat
Norway	The Norwegian AMR surveillance program (NORM)	The Norwegian Ministry of Health and Social Affairs	Humans, animals
Japan	The Japanese Veterinary Antimicrobial Resistance Monitoring Program (JVARM)	Ministry of Agriculture, Forestry and Fisheries	Animals

*^†^In collaboration with the United States Department of Agriculture (USDA) and the Center for Disease Control and Prevention (CDC).*

Coordination between the various stakeholders is paramount for effective surveillance systems at the country level. In Canada, a new initiative to better manage the dissemination of antimicrobial resistance at the human-animal interface was established by the Public Health Agency of Canada in 2015. The aim of this program, called the Canadian Antimicrobial Resistance Surveillance System (CARSS), is to strengthen the coordination and integration of antimicrobial resistance and antimicrobial use activities and information in Canada and to consolidate surveillance from seven existing systems (Gelbrand et al., 2015).

#### Practical Conditions for the Reduction of Colistin Use on Farms

Governments should fund research to enhance our understanding of environmental and genetics factors that facilitate the development of infectious disease in food animals, and to examine alternative strategies for the use of antibiotics on farms. Financial assistance for farmers in the implementation of sustainable practices and interventions to prevent infections, such as sanitation, housing, improvement of nutritional programs, and immunization, is very important for the reduction of the use of colistin or other antibiotics on farms. In addition, the preparation of guides and educational material for veterinarians and farmers on appropriate disease management and treatment based on the recent results of research is crucial for the responsible use of antimicrobials in farms. Efforts to improve microbiological laboratories are vital to help veterinarians undertake rapid therapeutic action with the most appropriate antibiotic and at an early stage of the disease (Årdal et al., 2009). Finally, the competent authorities should clearly define guidelines for colistin marketing, sales, and use on farms.

### Action in the Environment

In addition to the isolation of colistin resistant bacteria from manure, water, migratory birds, and vegetables (Hölzel et al., 2010; Schwarz and Johnson, 2016), the toxicity impact of colistin on the environment is a topic of concern (Bressan et al., 2013; Guo et al., 2014). Indeed, it has been shown that the presence of colistin at therapeutic concentrations in swine farm wastewater was associated with a toxicity against ammonia-oxidizing bacteria (AOB) (Bressan et al., 2013). These AOB are involved in the biodegradation of xenobiotic compounds and in the conversion of ammonia to nitrites in wastewater treatment plants (Bressan et al., 2013). The ecotoxicity effect of colistin was demonstrated in the earthworm *Eisenia fetida*; colistin caused significant damage to its intestinal epithelium and caused the induction of stress-related gene expressions (Guo et al., 2014).

In addition, it has been reported that colistin-resistant *E. coli* were isolated from wild rabbits (*Oryctolagus cuniculus*) and wild hares (*Lepus europaeus*) that have not been previously treated with colistin (Dotto et al., 2014). Consequently, wildlife may represent another potential reservoir of colistin resistance bacteria in the environment that could contaminate humans through contaminated food and water or by direct human and animal contact (Gelbrand et al., 2015).

This section will be devoted to the possible interventions to limit the spread of colistin resistant bacteria and genes in the environment via pig manure (**Figure 3**).

#### Reducing the Use of Antibiotics on Farms

It has been estimated that about 75% of the administered antibiotics is not absorbed by animals but is excreted via the feces or urine (Chee-Sanford et al., 2009). This finding is even more pronounced with colistin, which is very poorly absorbed in animal’s gastrointestinal tract (Rhouma et al., 2016a). It has also been reported that the frequency of bacteria carrying antimicrobial resistance genes is high in pig manure compared to other farm animals (Heuer et al., 2011), and a high frequency and concentration of ARGs was detected around swine farms (Chen et al., 2010). Therefore, the role of pig manure is not to be underestimated in the dissemination of colistin resistance in the environment. It is crucial to consider reducing the use of antibiotics on farms, especially critically important antimicrobials, in favor of other measures such as the improvement of nutritional programs, housing, and animal immunization (Pruden et al., 2013).

#### Biological Management of Manure

Some studies have reported that composting eliminates on average 50–70% of some antimicrobials such as chlortetracycline, monensin, and tylosin (Pruden et al., 2013) and reduces the relative quantities of the *bla*_TEM_, *sul3*, and *erm*(B) genes in manure (Le Devendec et al., 2015). However, to the best of our knowledge, no study has shown the efficacy of this technique in reducing amounts of colistin or *mcr* genes in pig manure.

The effectiveness of reducing ARGs in pig manure depends mostly on the method manure is handled; aerobic biofiltration of manure has been reported to reduce *erm*(X) more effectively than other ARG such as *erm*(F), *erm*(B), and *tet*(G), while mesophilic anaerobic digestion and lagoon storage reduced none of these AR genes (Chen et al., 2010). There has been much controversy concerning the efficiency of these biological manure treatments, such as lagoons and composting, in ARG reduction (Pruden et al., 2013), which is why more research is needed into assessing the effectiveness of swine waste treatment processes in the destruction of resistant bacteria and ARG in pig manure. With the lack of regulation worldwide or international guidelines to control the release of pig manure containing antibiotics (Wei et al., 2011), it is difficult to reduce the spread of colistin resistance into the environment by manure land applications.

### Action in Human Medicine

Colistin is currently considered to be one of the last-resort antibiotics used for the treatment of infections caused by MDR-GNB in humans (Bergen et al., 2015a). Maintaining the effectiveness of this antibiotic is a challenge for both scientists and physicians. Nevertheless, there are several possible proposals to optimize the use of colistin in human medicine (**Figure 3**).

#### Screen for Colistin Resistance in Patients

This step is crucial before undertaking a therapeutic intervention using colistin, and screening should be done in both patients with and without prior history of colistin usage (Olaitan et al., 2016b). Hospitals should know whether or not their laboratories have the ability and the necessary equipment to perform colistin resistance testing and *mcr-1* screening tests among admitted patients who needed colistin as a treatment.

#### Prevention of Contamination by Colistin Resistant Bacteria in Hospital

Hand hygiene plays a crucial role in achieving this goal (Mathur, 2011). Interactive educational programs are important to explain the steps of hand hygiene technique as well as its rationale. Given the coproduction of *mcr-1* genes and NDM enzymes by the same colistin resistant isolates, as reported by Du et al. (2016), we believe that the guide for the control of healthcare-associated infections due to carbapenem-resistant *Enterobacteriaceae*, published in 2012 by the USA Centers for Disease Control and Prevention (CDC) and updated in 2015 (available from: http://www.cdc.gov/hai/organisms/cre/cre-toolkit/index.html), would be a very good tool to prevent contamination by colistin resistant strains in hospitals. In addition, the identification of a patient carrying isolates that produce *mcr-1* gene in association with carbapenemases should be strictly considered a reason for patient isolation (Nordmann and Poirel, 2016).

#### Prevention of Contamination of Humans Following Direct Contact with Animals or Meat

Epidemiological studies have described a possible horizontal transmission of a colistin resistant *E. coli* strains from pigs (Olaitan et al., 2015) or from companion animals (Zhang, 2016) to humans following close contact. It has been shown that colistin-resistant *E. coli* was isolated from healthy individuals without prior colistin usage (Olaitan et al., 2016b). Better hygiene, particularly hand washing with soap or using alcohol disinfectant after handling animals at a farm, pet shop, or slaughterhouse is obligatory. Also, using gloves during pig or manure handling and taking a shower at the exit of a piggery are mandatory practices that should be enforced. As well, employees must be particularly familiar with hand hygiene techniques and their purpose. Considering that a high percentage of colistin resistant *E. coli* is isolated from retail meat (Liu et al., 2016), consumers should avoid any type of cross contamination between meat and salad or other raw foods.

#### Re-evaluation of Colistin Use for Selective Digestive Decontamination

In the intensive care unit, colistin is sometimes used orally for selective decontamination of the digestive tract (SDD), mainly to target resistant gram-negative aerobic bacteria, along with a short course of a parenteral broad-spectrum antimicrobial such as cefotaxime (a third generation cephalosporin) (Silvestri et al., 2007). This practice has been shown through meta-analysis of randomized control trials to reduce the occurrence of respiratory tract infections, mortality, and overall bloodstream infections in critically ill patients (De Jonge et al., 2003; Silvestri et al., 2007). However, it has been demonstrated that prolonged use of colistin as part of SDD is associated with the emergence of colistin resistance among ESBL producing *K. pneumoniae* isolates (Halaby et al., 2013). The long-term effects of colistin use in SDD was singled out as a possible source of colistin resistance amplification, therefore the re-evaluation of this practice is a topic of concern for intensive care units (Rawson et al., 2016).

#### Evaluation and Optimization of Colistin Combination Therapy

Several *in vitro* and in mouse model studies have shown that combination of colistin with other antimicrobials such as rifampicin and imipenem may be more effective than colistin monotherapy in the treatment of MDR-GNB (Årdal et al., 2009; Lagerbäck et al., 2016). A review of 15 studies involving 55 unique patient cases found that clinical success was lower for colistin monotherapy compared with colistin combination therapy for treatment of infections caused by *K. pneumoniae* carbapenemases (KPCs) producers (Hirsch and Tam, 2010). However, another review reported considerable controversy regarding the clinical efficacy of colistin combination therapy during the treatment of MDR-GNB (Tamma et al., 2012). This interesting therapeutic approach needs to be clinically studied in depth to assess its effectiveness and its impact in MDR-GNB resistance occurrence.

## Conclusion

Colistin is an antibiotic widely used in pigs for the oral control of bacterial infections caused by *E. coli* and *Salmonella*. The recent discovery of a plasmid-mediated *mcr-1* gene encoding for colistin resistance in *Enterobacteriaceae* has generated great concern about the possible loss of effectiveness of colistin for the treatment of MDR-GNB in humans. Because of the large amounts of colistin used in food animals and particularly in pigs, pig production has been pointed to as the greatest cause of colistin resistance amplification and spread. Consequently, experts, scientists, and government agencies have called for a reduction of colistin use in pigs and stressed that this antibiotic should be used only for the treatment of diseased animals as a last-resort treatment under strict circumstances. The *mcr-1* gene has been isolated on four continents from sources other than food animals, such as the environment and human origins, and some *E. coli* isolates carrying a plasmid-encoded *mcr-1* gene were associated with ESBL or carbapenemases enzymes. This highlights the need for an overarching approach on the judicious use of all antibiotics, especially those of critical importance for human health. The One Health concept is more important than ever to better manage colistin resistance at the human- animal-environment interface through the use of adequate science-based risk management policies that respect interdisciplinary regulations. Finally, we should start thinking beyond colistin therapy in swine and begin evaluating the effectiveness of other alternative strategies against infections caused by *Enterobacteriaceae*.

## Author Contributions

MR conceived and designed the study, wrote the paper; FB designed the study, revised the paper; WT designed the study, revised the paper; AL designed the study, participated in the drafting of the paper.

## Conflict of Interest Statement

The authors declare that the research was conducted in the absence of any commercial or financial relationships that could be construed as a potential conflict of interest.
